# Supplementary material for: Identification of Transcription Factors Responsible for a Transforming Growth Factor-β-Driven Hypertrophy-like Phenotype in Human Osteoarthritic Chondrocytes
Source: Cells. 2022 Apr 5;11(7):1232. doi: 10.3390/cells11071232 (PMC8998018; doi:10.3390/cells11071232)
Supplement: Supplementary file 1 [file cells-11-01232-s001.zip › cells-1634175-final SM.pdf]

Table S1. Full promoter sequences containing transcription factor elements (**bold**) driven by a minimal promoter including a TATA-box (underlined).

Pathway	Binding site	Sequence
MAPK/JNK	AP1	***TGAGTCAGTGACTCAGTGAGTCAGTGACTCAGTGAGTCAGTGACTCAG****CTCGAGGATATCAAGATCTGGCCTCGGCGGCCAAGCTTAGACACTAGAGGGTATATAATGGAAGCTCGACTTCCAG*
SOX9	SOX9	***AGAACAATGGAGAACAATGGAGAACAATGGAGAACAATGGAGAACAATGGAGAACAATGGAGAACAATGG****CTCGAGGATATCAAGATCTGGCCTCGGCGGCCAAGCTTAGACACTAGAGGGTATATAATGGAAGCTCGACTTCCAG*
NFκB	NFκB	***GGGAATTTCCGGGGACTTTCCGGGAATTTCCGGGGACTTTCCGGGAATTTCC****AGATCTGGCCTCGGCGGCCTAGATGAGACACTAGAGGGTATATAATGGAAGCTCGACTTCCAG*
Notch	CSL	***CGTGGGAAAATGGGCGGAAGGGCACCGTGGGAAAATAGTTGATCCCGACTCGTGGGAAAATGGGCGGAAGGGCACCGTGGGAAAATAGTTGATCCCGACTCGTGGGAAAAGGGCGGAAGGGCACCGTGGGAAAATAGTTGATCTGATGTACAAGATCCCGACTCGTGGGAAAATGGGCGGAAGGGCACCGTGGGAAAATAGTTGATCCCGACTCGTGGGAAAATGGGCGGAAGGGCACCGTGGGAAAATAGTTGATCCCGACTCGTGGGAAAAGGGCGGAAGGGCACCGTGGGAA****CTCGAGGATATCAAGATCTGGCCTCGGCGGCCAAGCTTAGACACTAGAGGGTATATAATGGAAGCTCGACTTCCAG*
cAMP/PKA	CRE	***TGACGTCAGCTGCCAGATCCCATGGCCGTCATACTGTGACGTCTTTCAGACACCCCATTGACGTCAATGGGAGAAC****AGATCTGGCCTCGGCGGCCAAGCTTAGACACTAGAGGGTATATAATGGAAGCTCGACTTCCAG*
Glucocorticoid signaling	GRE	***AGAACATTTTGTCCGAGAACATTTTGTCCGAGAACATTTTGTCCGAGAACATTTTGTCCGAGAACATTTTGTCCGAGAACATTTTGTCCG****CTCGAGGATATCAAGATCTGGCCTCGGCGGCCAAGCTTAGACACTAGAGGGTATATAATGGAAGCTCGACTTCCAG*
TGF-β	SBE	***AGTATGTCTAGACTGAAGTATGTCTAGACTGAAGTATGTCTAGACTGA****CTCGAGGATATCAAGATCTGGCCTCGGCGGCCTAGATGAGACACTAGAGGGTATATAATGGAAGCTCGACTTCCAG*
Wnt	TCF/LEF	***AGATCAAAGGGTTTAAGATCAAAGGGCTTAAGATCAAAGGGTATAAGATCAAAGGGCCTAAGATCAAAGGGACTAAGATCAAAGGGTTTAAGATCAAAGGGCTTAAGATCAAAGGGCCTA****CTCGAGGATATCAAGATCTGGCCTCGGCGGCCAAGCTTAGACACTAGAGGGTATATAATGGAAGCTCGACTTCCAG*
INF-α	ISRE	***TAGTTTCACTTTCCCTAGTTTCACTTTCCCTAGTTTCACTTTCCCTAGTTTCACTTTCCCTAGTTTCACTTTCCC****CTCGAGGATATCAAGATCTGGCCTCGGCGGCCAAGCTTAGACACTAGAGGGTATATAATGGAAGCTCGACTTCCAG*
IL-6	SIE	***AGCTTCATTTCCCGTAAATCGTCGAAGCTTCATTTCCCGTAAATCGTCGAAGCTTCATTTCCCGTAAATCGTCGAAGCTTCATTTCCCGTAAATCGTCGAAGCTTCATTTCCCGTAAATCGTCGA****CTCGAGGATATCAAGATCTGGCCTCGGCGGCCAAGCTTAGACACTAGAGGGTATATAATGGAAGCTCGACTTCCAG*
MAPK/ERK	SRE	***AGGATGTCCATATTAGGACATCTAGGATGTCCATATTAGGACATCTAGGATGTCCATATTAGGACATCTAGGATGTCCATATTAGGACATCTAGGATGTCCATATTAGGACATCT****AGATCTGGCCTCGGCGGCCAAGCTTAGACACTAGAGGGTATATAATGGAAGCTCGACTTCCAG*
RhoA	SRF	***AGTATGTCCATATTAGGACATCTACCATGTCCATATTAGGACATCTACTATGTCCATATTAGGACATCTTGTATGTCCATATTAGGACATCTAAAATGTCCATATTAGGACATCT****AGATCTGGCCTCGGCGGCCAAGCTTAGACACTAGAGGGTATATAATGGAAGCTCGACTTCCAG*
Oxidative stress	ARE	***TAGCTTGGAAATGACATTGCTAATGGTGACAAAGCAACTTTTAGCTTGGAAATGACATTGCTAATGGTGACAAAGCAACTTT****CTCGAGGATATCAAGATCTGGCCTCGGCGGCCAAGCTTAGACACTAGAGGGTATATAATGGAAGCTCGACTTCCAG*
Hyperosmotic signaling	NFAT5	***TGGAAAAGTCCATGGAAAAGTCCATGGAAAAGTCCATGGAAAAGTCCATGGAAAAGTCCATGGAAAAGTCCATGGAAAAGTCCATGGAAAAGTCCA****CTCGAGGATATCAAGATCTGGCCTCGGCGGCCAAGCTTAGACACTAGAGGGTATATAATGGAAGCTCGACTTCCAG*
PPARγ	PPRE	***GTCGACAGGGGACCAGGACAAAGGTCACGTTCGGGAGTCGACAGGGGACCAGGACAAAGGTCACGTTCGGGAGTCGAC****CTCGAGGATATCAAGATCTGGCCTCGGCGGCCAAGCTTAGACACTAGAGGGTATATAATGGAAGCTCGACTTCCAG*

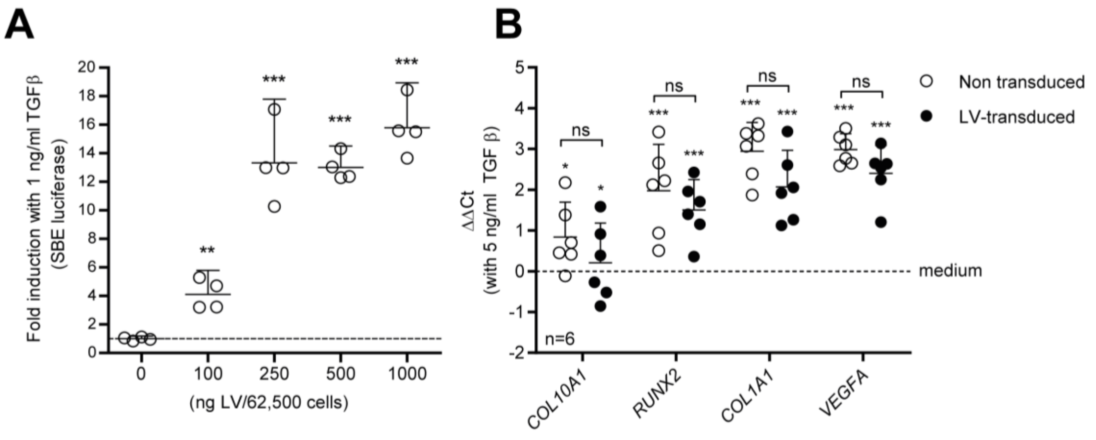

Figure S1. Primary human OA chondrocytes can be efficiently transduced with lentivirus, thereby not affecting TGF-β1-induced signaling. To identify which transcription factors (TF) were activated by TGF-β1 in primary human OA chondrocytes, we made use of luciferase-based TF activity essays. Binding sequences specific for 15 different transcription factors extended with a minimal promoter were directionally cloned into the pNL1.2 vector. Subsequently, lentiviral constructs were generated and in Lenti-X 293T cells viral supernatants were produced using 1 mg/ml polyethylenimine (PEI). Viral supernatant was concentrated and lentiviral concentration was determined using p24 ELISA assay. (**A**) First, transduction protocol was optimized in primary human OA chondrocytes using lentivirus (LV) containing the SBE-pNL1.2 luciferase construct. Human OA chondrocytes (n=1), cultured for a week to form a monolayer, were transduced for 6-8 hours with different concentrations LV (0, 100, 250, 500 and 1000 ng) per 62,500 cells. After 48 hours the transduced cells were stimulated with 1 ng/ml TGF-β1 for 6 hours. Luminescence was measured at 470-480 nm and fold induction compared to medium-stimulated cells was calculated. Each condition was performed in quadruple and 95% CI was depicted. (**B**) Using qPCR we checked if lentiviral transduction with 500 ng LV/62,500 cells did not affect running of our TGF-β-driven chondrocyte hypertrophy-like model. Stimulation for 48 hours with 5 ng/ml TGF-β resulted in increased gene expression of *COL10A1*, *RUNX2*, *COL1A1* and *VEGFA* in both non transduced (white dots) and LV-transduced (black dots) OA chondrocytes (n=6). Data are plotted as mean ± 95% CI with each dot representing the average of three technical replicates in one chondrocyte donor. Statistical analysis was performed using repeated measures one-way ANOVA with Bonferroni's post hoc test: ns = non-significant; * $p \leq 0.05$; ** $p \leq 0.01$; *** $p < 0.001$.

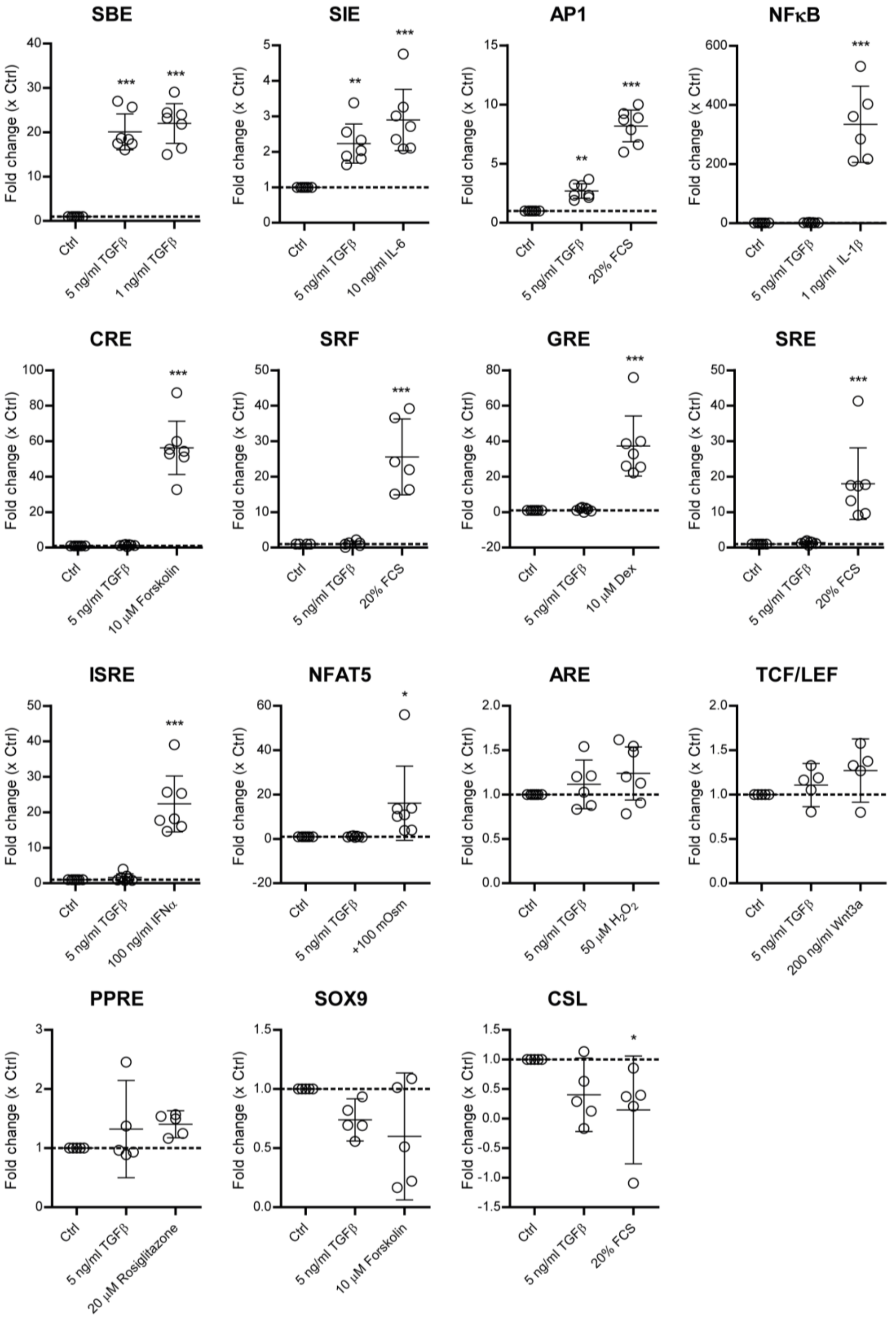

Figure S2. Investigation of transcription factor activation by 5 ng/ml TGF-β1 in primary human OA chondrocytes. To identify which transcription factors (TF) were activated by TGF-β1 in primary human OA chondrocytes, we made use of luciferase-based TF activity essays. Binding sequences specific for 15 different transcription factors (SBE, SIE, AP1, NFκB, CRE, SRF, GRE, SRE, ISRE, NFAT5, TCF/LEF, PPRE, SOX9, CSL and ARE) extended with a minimal promoter were directionally cloned into the pNL1.2 vector. Subsequently, lentiviral constructs were generated and in Lenti-X 293T cells viral supernatants were produced using 1 mg/ml polyethylenimine (PEI). Viral supernatant was concentrated and lentiviral concentration was determined using p24 ELISA assay. Primary human OA chondrocytes, cultured for a week to form a monolayer, were transduced for 6-8 hours with 500 ng of lentivirus per 62,500 cells. After 48 hours the transduced cells were stimulated with

a positive control, and with 5 ng/ml TGF-β1 for 6 hours. Luminescence was measured at 470-480 nm and fold change compared to medium-stimulated cells (Ctrl) was calculated. Data are plotted as mean ± 95% CI with each dot representing the average of a quadruple sample in one chondrocyte donor. Statistical analysis was performed using an repeated measures one-way ANOVA with Bonferroni's post hoc test: * ≤ 0.05; ** p ≤ 0.01; *** p < 0.001.

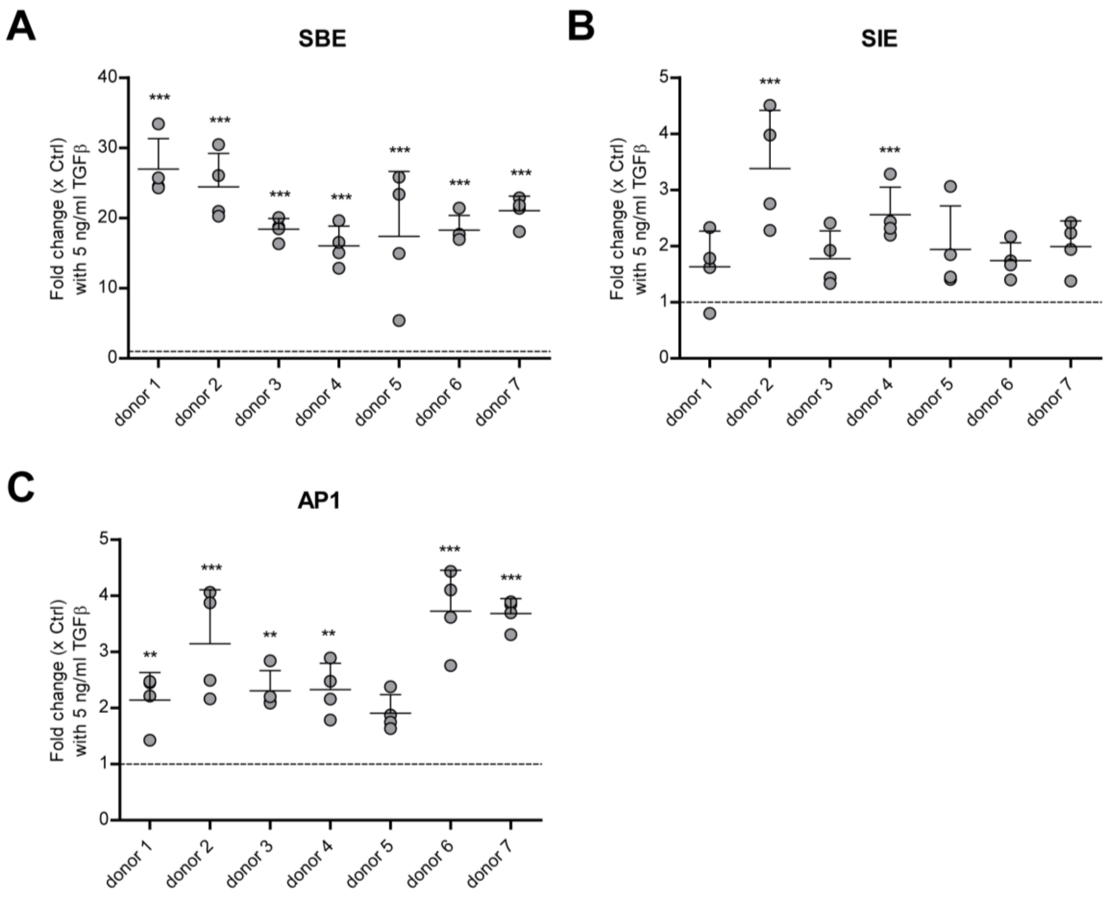

Figure S3. TGF-β1 activates SMAD3:4, STAT3 and AP1 signaling in human OA chondrocytes. Primary human OA chondrocytes (n=7), cultured for a week to form a monolayer, were transduced for 6-8 hours with 500 ng of lentivirus per 62,500 cells. After 48 hours the transduced cells were serum-starved overnight and then stimulated with a positive control, and with 5 ng/ml TGF-β1 for 6 hours. Luminescence was determined at 470-480nm and fold change compared to medium-stimulated cells was calculated. TGF-β1 activated cell signaling regulated by the transcription factors (**A**) SMAD3:4 (SBE: SMAD Binding Element), (**B**) STAT3 (SIE: Interleukin (IL)-6 sis-inducible element or STAT3 response element) and (**C**) AP1 (Activation Protein 1). The fold change with 5 ng/ml TGF-β1 is depicted per donor (quadruple sample ± 95% CI). Statistical analysis was performed using an one-way ANOVA with Bonferroni's post hoc test: ** p ≤ 0.01; *** p < 0.001.

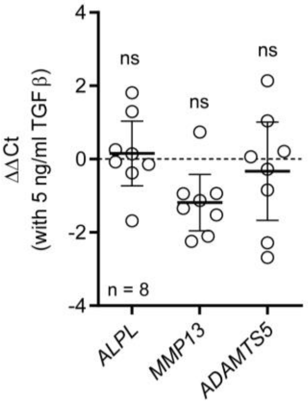

Figure S4. Late-hypertrophic genes *ALPL*, *MMP13* and *ADAMTS5* are not increased by TGF-β1 in primary human OA chondrocytes. Human chondrocytes of eight donors were cultured in monolayer and stimulated with 10 ng/ml TGF-β1 48 hours. No significant differences were observed on relative *alkaline phosphatase* (*ALPL*), *matrix metallopeptidase 13* (*MMP13*) and *ADAM Metallopeptidase with thrombospondin type 1 motif 5* (*ADAMTS5*) expression, all late-hypertrophic genes. Data are plotted as mean ± 95% CI with each dot representing the average of three technical replicates in one chondrocyte donor. Statistical analysis was performed using an one-way ANOVA with Bonferroni's post hoc test: ns = non-significant.
